# Evaluation of novel chromatographic prototypes for supercoiled plasmid DNA polishing

**DOI:** 10.3389/fbioe.2023.1296444

**Published:** 2024-01-04

**Authors:** Pedro L. Ferreira, Helena Marie, Tim Berger, Bianca Edelmann, Oliver Rammo, Fani Sousa

**Affiliations:** ^1^ CICS-UBI—Health Sciences Research Centre, University of Beira Interior, Covilhã, Portugal; ^2^ Merck Life Science KGaA, Darmstadt, Germany

**Keywords:** agmatine-based resin, arginine-based resin, chromatographic polishing, plasmids purification, supercoiled plasmid DNA

## Abstract

Since the world first approved gene therapeutics, nucleic acid-based therapies have gained prominence. Several strategies for DNA-based therapy have been approved, and numerous clinical trials for plasmid DNA (pDNA)-based vaccines are currently in development. Due to the rising interest in pDNA for vaccination and gene therapy, plasmid manufacturing must become more effective. One of the most critical steps is downstream processing, involving isolation and purification procedures. To comply with the regulatory guidelines, pDNA must be available as a highly purified, homogeneous preparation of supercoiled pDNA (sc pDNA). This process undertakes several challenges, primarily due to the diversity of molecules derived from the producer organism. In this study, different resins were tested for the adsorption and selective polishing of sc pDNA. To identify optimal pDNA adsorption conditions, batch and column assays were performed with different resins while promoting electrostatic and hydrophobic interactions. The effect of ionic strength, pH, and contact time were evaluated and optimized. Additionally, static and dynamic binding capacities were determined for the selected resins. Analytical chromatography and agarose gel electrophoresis were used to assess the selectivity of the most promising resins toward sc pDNA isoform. Also, genomic DNA, endotoxins, and proteins were quantified to characterize the final sc pDNA quality. At the same time, the recovery and purity yields were evaluated by quantification of sc pDNA after the purification procedure. Overall, the results of the chromatographic assays using agmatine- and arginine-based resins have shown promising potential for sc pDNA polishing. Both resins demonstrated excellent binding capacity for pDNA, with agmatine outperforming arginine-based resin in terms of capacity. However, arginine-based resin exhibited a superior pDNA recovery yield, reaching a notable 52.2% recovery compared to 10.09% from agmatine. Furthermore, both resins exhibited high relative purity levels above 90% for the sc pDNA. The comprehensive characterization of the recovered sc pDNA also revealed a significant reduction in gDNA levels, reinforcing the potential of these prototypes for obtaining high-quality and pure sc pDNA. These findings highlight the promising applications of both resins in scalable pDNA purification processes for gene therapy and biopharmaceutical applications.

## Introduction

The significance of pDNA cannot be underestimated in developing DNA vaccines and gene therapy products (Lopes et al., 2019; Sung and Kim, 2019). Its potential to revolutionize the field of medicine has been demonstrated, and from the more than 2500 clinical trials conducted since the initial gene therapy trial, nearly 20% have effectively utilized pDNA (Anguela and High, 2019). Recent breakthroughs in using pDNA to develop DNA vaccines to fight SARS-CoV-2 are particularly noteworthy (Lassaunière et al., 2021). The increased interest in pDNA, demands new developments in downstream processing technologies to have higher yields of pharmaceutical-grade products, resulting in more cost-effective and safer biopharmaceuticals (Schmeer et al., 2017).

After biosynthesis in *E. coli* (*Escherichia coli*), several steps are required to isolate and purify pDNA. The starting material for pDNA purification is usually a lysate derived from alkaline lysis, which has a complex composition of no more than 3% of pDNA (Sousa et al., 2010a). In comparison, 97% represent other impurities, namely, RNA, genomic DNA (gDNA), endotoxins, open circular pDNA (oc pDNA), and proteins. Most critical impurities share some characteristics with pDNA, meaning they are negatively charged and similar in size and hydrophobicity (Prazeres and Monteiro, 2014). This represents the biggest challenge for pDNA purification and application. As such, the efficiency of the whole process is of the utmost importance, as the impurities can cause immune reactions and reduce the efficacy of the therapy or vaccine.

Regulatory agencies, such as the U.S. Food and Drug Administration and the EMEA (European Agency for the Evaluation of Medical Products), recommend that a final pDNA therapeutic product should meet the strict quality criteria described in Table 1, and the absolute bulk should have more than 90% of the pDNA in the supercoiled isoform state (Urthaler et al., 2012; Valente et al., 2021; Ferreira et al., 2023).

**TABLE 1:** Suggested criteria for pDNA-based vaccines from regulatory agencies, FDA (Food and Drug Administration) and EMA (European Medicines Agency).

Impurities	Specifications	
Proteins	Undetectable	
gDNA	<1%	2 µg gDNA/mg pDNA
RNA	Undetectable	
Endotoxins	40 EU/mg pDNA	
Microorganisms	<1 CFU	

Large-scale purification of pDNA is commonly based on chromatographic techniques, which offer high resolution and cover a wide range of different modalities (Prazeres, 2011). Various methods, including size-exclusion, reversed-phase, hydrophobic interaction, and affinity chromatography, have been used for pDNA purification (Sousa et al., 2010b; Carapito et al., 2023). Notably, ion-exchange chromatography is the most employed method, facilitated by the fast binding between the negative charged phosphate groups in pDNA and positively charged groups on anion exchange resins (Guerrero-Germán et al., 2011). The selection of different chromatography modalities allows for a comprehensive exploitation of pDNA properties, such as, size, charge, hydrophobicity, and accessibility to nucleotide bases, enabling tailored purification strategies. However, conventional chromatographic resins still pose limitations regarding the purification of pDNA, not only due to the characteristics and similarities of impurities, but also associated with limitations inherent in available stationary phases (Sousa et al., 2008b). Due to pDNA size, using traditional chromatographic resins leads to slow mass transfer and low binding capacity since these resins were initially developed for protein purification (Urthaler et al., 2005; Tarmann and Jungbauer, 2008). Additionally, the larger size of pDNA increases pressure drops and leads to long processing times, low resolution of isoforms, low recovery, and potential fouling (Borujeni and Zydney, 2014; Valente et al., 2021).

Therefore, there is a need to find new and improved chromatographic resins to tackle pDNA purification, distinguish pDNA isoforms and isolate the supercoiled pDNA biologically active topology, attenuating the current limitations of this step.

## Materials and methods

### Materials

All chemicals and reagents used were of analytical grade. The prototype resins used for purification screening were synthesized by Merck KGaA (Darmstadt, Germany), and their chemistry is described in Table 2. Sodium chloride was purchased from Panreac (Barcelona, Spain), and tris(hydroxymethyl) aminomethane (Tris) from Merck (Darmstadt, Germany). L-Arginine used in the elution buffer was purchased from Thermo Fisher Scientific (Massachusetts, United States). All solutions were prepared using ultra-pure grade water purified with a Milli-Q system from Merck Millipore (Darmstadt, Germany). QIAGEN Plasmid Mega Kit was purchased from Qiagen (Hilden, Germany). Econo-Pac chromatography columns used in the gravity columns assays were purchased from Bio-Rad (California, United States).

**TABLE 2:** Prototype general description, chemistry, matrix, particle size, and ionic capacity.

Prototype	Ligand Chemistry	Matrix	Particle size (µm)	Ionic capacity (µeq/g resin)
Prototype 1	Agmatine	Polymeric bead	50	691
Prototype 14	Arginine	Polymeric bead	50	200

## Methods

### Bacterial growth and pDNA production

pDNA was obtained from an *E. coli* DH5α strain culture, previously transformed with a plasmid with 7.905 kb and pcDNA3-myc-FLNa S2152A (14.086 kb) (Addgene plasmid # 8983; http://n2t.net/addgene:8983; RRID: Addgene_8983). Bacterial growth was carried out at 37°C using Terrific Broth medium (12 g/L tryptone, 24 g/L yeast extract, 4 mL/L glycerol, 0.017 M KH_2_PO_4_, and 0.072 M K_2_HPO_4_) supplemented with 100 μg/mL ampicillin. The growth was kept for 16 h for optimal pDNA production. Cells were recovered by centrifugation at 3900 *g*, for 10 min, at 4°C, and stored at −20°C.

### pDNA extraction and purification

For the extraction of pDNA to be used as control sample in the screening assays, the QIAGEN Plasmid Mega Kit (Qiagen, Germany) was used according to the protocol provided by the manufacturer. The protocol describes alkaline lysis followed by the binding of pDNA to an anion exchange resin, which is promoted under appropriate salt and pH conditions. Impurities are removed during the washing step, and pDNA is finally eluted with a high salt buffer, being then concentrated through isopropanol precipitation. The pDNA sample concentrations were determined with a NANOPhotometer (Implen) at 260 nm, and its integrity and purity were routinely assessed by 0.7% agarose gel electrophoresis.

### Batch assays

Batch assays were performed using various resins with different chemistries and characteristics to optimize and understand pDNA adsorption and elution. The method can be divided into three main steps: 1) the equilibration step, where 2 mg of each resin was equilibrated with an equilibration buffer, followed by 20 min of contact time, in agitation and posterior centrifugation at 8,000 *g* for 2 min to separate and remove the aqueous phase and recover the equilibrated resin. The pDNA samples were also diluted within the same equilibration buffer; 2) the adsorption step, where the sample (1 mL of 50 μg/mL of pDNA) was put in contact with the resin to promote the adsorption. The mixture was kept at room temperature (RT) (approximately 25°C) in agitation to allow nucleic acid adsorption to the resin. The adsorption was screened at different contact times (20, 40, 60, and 120 min). Then, another centrifugation at 8,000 *g* for 2 min was performed to separate the solid phase from the aqueous phase. The supernatant was recovered to analyze non-bound pDNA, regarding its concentration, integrity, and purity. 3) the elution step, where 1 mL of elution buffer was added to the resin, to revert the interactions established with the pDNA sample, allowing its recovery. Various elution conditions were explored, encompassing distinct contact times, of 20 min and 120 min. The mixture was then centrifuged at 8,000 *g* for 2 min, and the supernatant was collected for pDNA recovery analyses. pDNA concentration was determined by measuring the absorbance at 260 nm, with a NANOPhotometer (Implen). Variations to the adsorption/elution contact times, sample compositions, plasmid size, and buffers were assessed with changes to pH, salt type, and concentration, as outlined in more detail in the “Results” section.

### Adsorption isotherms

Adsorption isotherms were performed to determine the static binding capacity (SBC) and binding efficiency of each prototype. Aliquots containing a fixed amount of resin (2 mg) were incubated with varying concentrations of pDNA (5, 10, 20, 30, 40, 50, and 75 μg/mL). The resins were equilibrated with the pre-determined equilibration buffer. Thus, prototype 1 was tested with 50 mM Tris-HCl, pH 8.0, while prototype 14 was tested with 100 mM Tris-HCl, pH 8.0. A dilution series of each pDNA concentration was prepared in equilibration buffer, and 1 mL aliquots were added to the resin vials. The vials were gently agitated for 120 min to achieve equilibrium. After incubation, centrifugation at 8,000 *g* was performed for 2 min to separate and recover the aqueous phase. pDNA concentration in the decanted supernatant was measured at 260 nm. The Langmuir model was used, and experimental data were fit to Eq. 1, where *q* and *c* represent the respective stationary and mobile phase pDNA concentration, *q*_*max*_ is the maximum binding capacity at equilibrium, and *K*_*L*_ is the Langmuir constant.

$$q=\frac{q_{\max}*K_{L}*c}{1+K_{L}*c}$$ (1)

### Gravity column assays

**FIGURE 1:**
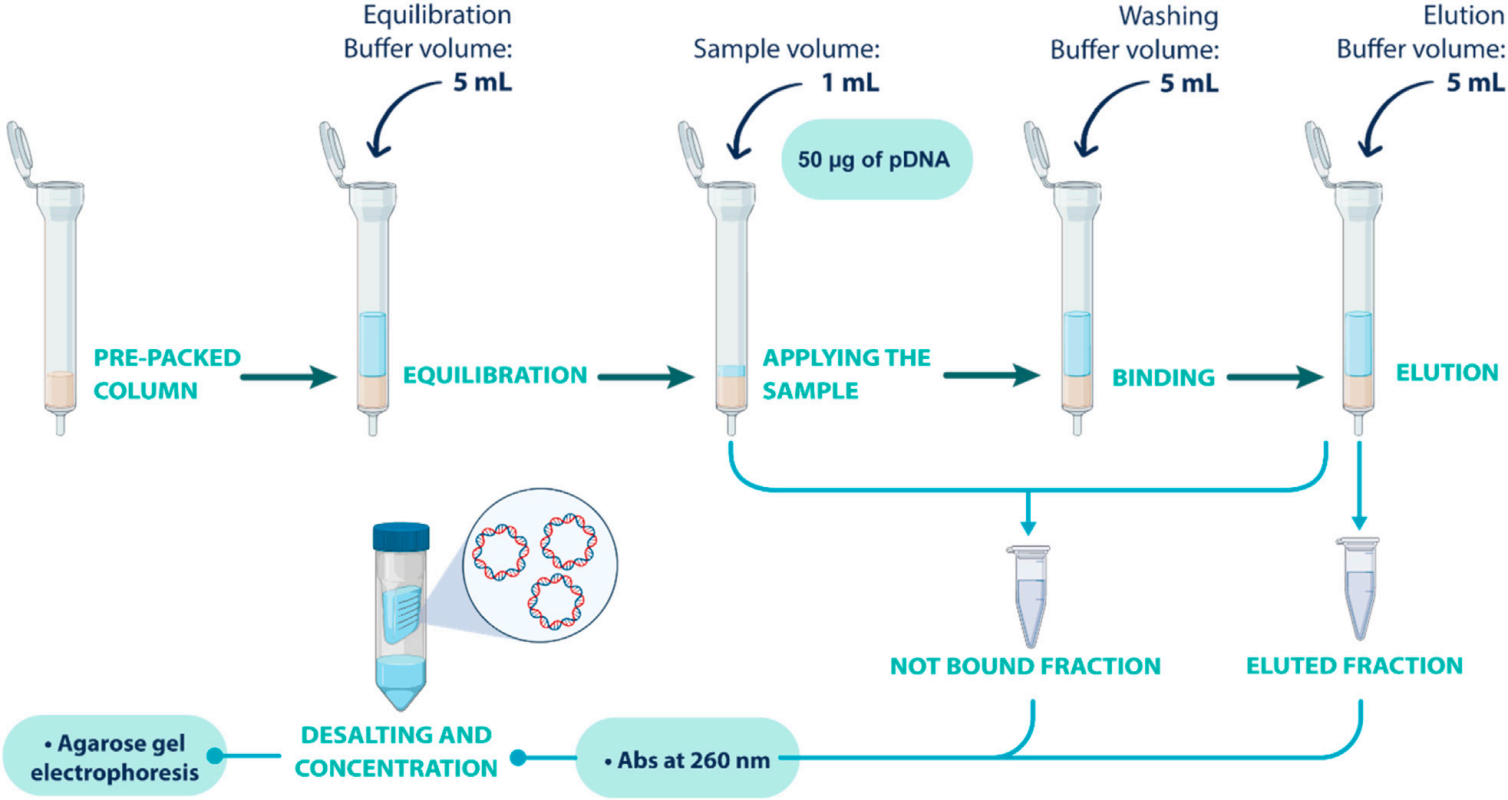
Schematic representation of the gravity column assays performed to screen the optimal conditions for separating oc and sc pDNA isoforms.

After optimizing adsorption/elution conditions in batch, the conditions for the selective separation of the oc pDNA and sc pDNA were optimized in column assays. For that, 100 mg of each resin was packed in gravity-derived flow columns. Equilibration buffer (5 mL) was added to promote the desired interactions between pDNA and the resin. Then, 1 mL of pDNA sample (50 µg of pDNA) was added to the column, followed by a wash step with binding buffer (5 mL). Finally, elution buffer (5 mL) was added to elute the pDNA sample. Non-bound fractions were collected after sample application until the elution step and analyzed, while the eluted fractions were recovered after the elution step. After recovering both fractions, a desalting and concentration step was performed to remove the salt from the buffers. This process aimed to mitigate the interference in subsequential analytical steps and ensure the accuracy of the analyzes. Vivaspin^®^610,000 MWCO PES, manufactured by Sartorius (Gottingen, Germany) were used. Following sample application, a centrifugation was performed at 1100 g for 30 min. The concentrator was then refilled with the exchange buffer and a subsequent centrifugation cycle was executed. Post-centrifugation, the retentate samples were adjusted to their initial applied volume, unless concentration was necessary. The schematic procedure described here is illustrated in Figure 1.

### Chromatographic assays

Chromatographic assays were performed in an ÄKTA™ Avant chromatography system (Cytiva). A 16 mm diameter × 200 mm long empty column (XK 16/20 column from Cytiva) was individually packed. The column bed volume for each resin was precisely adjusted to 1 mL, facilitating consistent and reproducible results between optimization assays. Since the final goal of these resins wherein tested is to be employed in polishing chromatography, the sample used in these assays was derived from a complex *E. coli* lysate after a capture step performed by Merck KGaA. The sample was obtained after *E. coli* cells were subjected to lysis under alkaline conditions, releasing the plasmid. Subsequently, the lysate underwent filtration to remove cellular debris, followed by the addition of a conditioning buffer to prepare the sample for loading. The sample was then immobilized on a Natrix^®^ Q Pilot membrane, employing an Equilibration buffer containing 1 M potassium acetate, 160 mM NaCl, pH 5.0. Finally, elution was carried out using a buffer consisting of 1 M NaCl in 100 mM Tris-HCl buffer, pH 9.0. The chromatographic runs were performed at room temperature (approx. 25°C), and conditions were optimized for each resin. In these studies, three different mobile phases were utilized. For prototype 1, mobile phase A (MPA) for equilibration and binding consisted of 150 mM NaCl in 100 mM Tris-HCl buffer, pH 8.0; mobile phase B (MPB) for the first elution step consisted of 1.5 mM NaCl, 375 mM arginine in 100 mM Tris-HCl buffer, pH 8.0; and mobile phase C (MPC) for the second elution step consisted of 3 M NaCl in 10 mM Tris-HCl buffer, pH 8.0. For prototype 14, MPA consisted of 100 mM Phosphate buffer, pH 6.0; MPB was composed of 35 mM arginine in 100 mM Tris-HCl buffer, pH 8.0; and as MPC was used 3 M NaCl in 10 mM Tris-HCl buffer, pH 8.0. The assays were performed at a flow rate of 1 mL/min, and the absorbance of the eluate was continuously monitored throughout the assay at 260 nm. The column was first equilibrated with MPA. pDNA samples were diluted in the same buffer (100 μg/mL) and were loaded onto the column using a 200 µL loop at the same flow rate. Following pDNA injection, a washing step was performed with MPA. Then, a first elution condition was established by increasing the ionic strength with MPB, as optimized in the gravity column assays. A second elution was performed with MPC, and the fractions were pooled according to the chromatograms obtained. The fractionated samples were concentrated and desalted using Vivaspin^®^610,000 MWCO PES from Sartorius (Gottingen, Germany) for further analytical evaluation.

### Protein analysis

According to the manufacturer’s instructions, the microassay procedure from Bio-Rad Protein Assay (Bio-Rad, California, United States) was used to determine the total protein concentration in the fractionated samples recovered after the chromatographic assays. A calibration curve was designed using bovine serum albumin (BSA) as a protein standard, with the linear range set between 0.05 and 0.5 mg/mL. A 10 mM Tris-HCl, pH 8.0 buffer was utilized to create the dilutions. Each standard and sample solution were quantified in triplicate, after desalting, using 10 μL of sample and 200 μL of diluted Dye Reagent Concentrate provided by the manufacturer (1 volume of dye reagent for 4 volumes of distilled deionized water). The absorbance of the samples was evaluated at 595 nm after 15 min of incubation at 37°C.

### gDNA analysis

For quantitative analysis of gDNA, real-time polymerase chain reaction (qPCR) was performed in 96-well optical plates, using the Maxima^®^ SYBR Green/Fluorescein qPCR Master Mix (2X) (Thermo Fisher Scientific Inc.) in a CFX Connect™ Real-Time PCR Detection System (BioRad). A calibration curve was established using gDNA concentrations that ranged from 0.005 to 50 ng/μL. The quantitation cycle (Cq) was then correlated with the logarithm of the corresponding gDNA concentrations to establish the calibration curve. For the qPCR reaction, specific primers for the 16S ribosomal RNA (rRNA) gene were used (forward primer - 5′-ACA​CGG​TCC​AGA​ACT​CCT​ACG-3′; and reverse primer - 5′-CCG​GTG​CTT​CTT​CTG​CGG​GTA​ACG​TCA-3′). The reaction conditions started at 95°C for 10 min to initiate denaturation, followed by 40 cycles of 95°C for 10 s, 60°C for 30 s, and 72°C for 15 s. Finally, the samples were incubated at 65°C for 5 s with a gradual increase of 0.5°C until reaching 95°C for the melting curves. All reactions were performed in triplicate, and Cq values were averaged.

### Endotoxin analysis

The level of endotoxin contamination was determined using the ToxinSensor™ Chromogenic Limulus Amoebocyte Lysate (LAL) Endotoxin Assay Kit from GenScript (United States). A calibration curve ranging from 0.1 to 1.0 EU/mL was performed using a 10 EU/mL *E coli* endotoxin stock solution included in the kit, according to the manufacturer’s instructions. The samples and the kit samples were diluted in non-pyrogenic water, which also served as the blank, to prevent interference from external endotoxins. Only endotoxin-free tubes and tips were used during the quantification process.

### pDNA quantification

The purity and recovery of pDNA were evaluated after the chromatographic assay from each resin. The TSKgel^®^ DNA-NPR 2.5 µm, 4. 6 mm ID × 7.5 cm column from Tosoh Bioscience GmbH (Germany) was utilized to achieve the task. The method was adapted from the manufacturer’s description, based on weak anion exchange chromatography, and was developed to characterize pDNA samples. All the assays were performed at a flow rate of 0.5 mL/min and at room temperature. The absorbance was continuously monitored throughout the assay at 260 nm. The first step was equilibrating the column using 45% of 20 mM Tris-HCl buffer, pH 9.0 (MPA). Then, 50 µL of the sample, diluted in the equilibration buffer, is injected. After sample injection, the column was washed in the same conditions of the equilibration step for 1 min, and then the gradient for elution was applied. To achieve the highest resolution between oc and sc pDNA, the gradient was established from MPA to MPB (65% of 1 M NaCl in 20 mM Tris-HCl buffer, pH 9.0) over 10 min. To quantify the sc pDNA, a calibration curve was established with pDNA standards, ranging from 2.5 μg/mL to 200 μg/mL, purified with QIAGEN Plasmid Mega Kit. The degree of purity was determined by calculating the percentage of the peak area of sc pDNA in relation to the total area. Also, to confirm and validate the oc pDNA peak’s identity, a pDNA sample was digested by the restriction enzyme Nt.BspQI from New England Biolabs, Inc. (United States of America) and injected in the same conditions, to compare the retention profiles.

### Dynamic binding capacity

For measuring the dynamic binding capacity (DBC) of the prototypes under study, it was used the same apparatus that was used during the chromatographic assays. This apparatus consisted of a XK 16/20 column from Cytiva, with 1 mL of packed resin, connected to an ÄKTA™ Avant chromatography system (Cytiva). These studies were conducted at a flow rate of 0.5 mL/min, at room temperature, and with a pDNA concentration of 25 μg/mL. The resins were equilibrated with the respective MPA, and the samples were prepared with the same ionic strength as the buffer. Determination of the DBC was carried out by recording the breakthrough curves and calculating the amount of bound pDNA per mL resin at 10% and 50% breakthrough. The bound pDNA was eluted by increasing the ionic strength using MPC. Finally, the resin was regenerated with a 0.2 M NaOH solution. Absorbance was continuously monitored throughout the assay at 260 nm.

## Results and discussion

The type of chromatographic support necessary for separating pDNA differs from that required for protein purification. When it comes to plasmids, their larger size means that they usually bind to functional groups on the surface of the resin. However, this means their binding capacity will be significantly lower than that of proteins. It is essential to consider the chemistry and ionic capacity of the materials used to ensure effective binding. Initially, a screening was performed for the evaluation of multiple prototypes (results not shown), exploiting a diverse array of interactions with sc pDNA with the goal of find the most promising candidates. The different prototypes cover aromatic adsorption utilizing functional groups like picolylamines and pyridinium, amino acid-DNA interactions using agmatine, arginine, histidine, histamine, leucine, and lysine as functional ligands, as well as hydrophobic interactions involving butyl functional chemistries. The first screening unveiled that the resins based on arginine and agmatine ligands, performed better, showing promise for sc pDNA purification. Consequently, this study presents the detailed investigation on the optimization and characterization of both prototypes based on arginine and agmatine, aiming to enhance the purification process for sc pDNA. Table 2 describes their chemistry, particle size, matrix, and ionic capacity.

## Batch assays

### Screening of adsorption conditions

**FIGURE 2:**
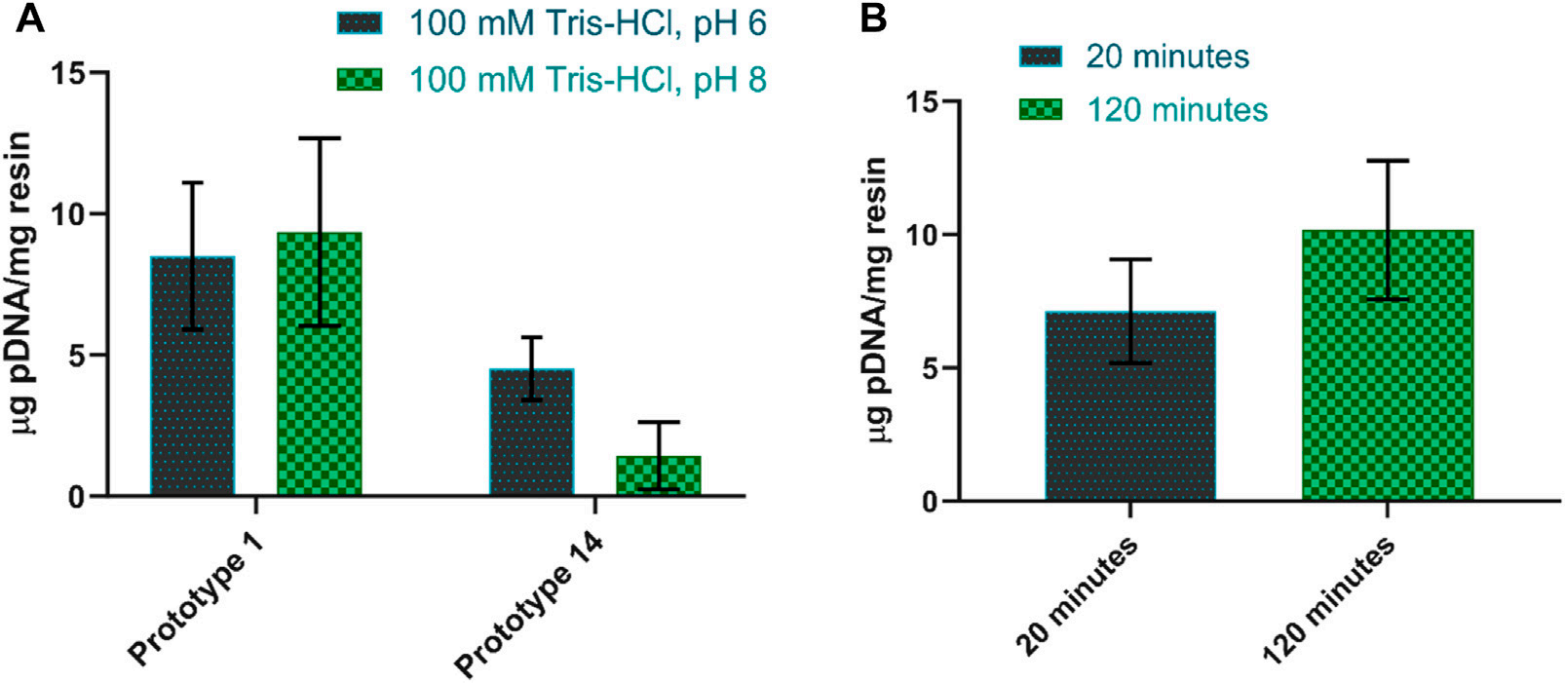
**(A)** Amount of pDNA adsorbed per mg of resin when using buffers at pH 6 and pH 8 for prototype 1 and prototype 14. **(B)** Amount of pDNA adsorbed per mg of resin when changing the contact time between 20 and 120 min for prototype 1. The equilibration buffer used was 100 mM Tris-HCl, pH 8.0.

Screening the adsorption conditions in batch allowed the identification of optimal parameters that enhance pDNA binding during chromatography, leading to improved yields. Furthermore, an in-depth adsorption behavior provides valuable insights into the interaction between pDNA and chromatography resins, facilitating the development of robust and scalable purification processes. Initially, two types of interactions were mainly promoted: electrostatic interactions, which occur under low salt conditions, and hydrophobic interactions, which occur under high salt conditions. When 1.5 M and 2.5 M ammonium sulfate were added to promote hydrophobic interactions, both prototypes (corresponding to arginine and agmatine chemistries) showed no binding (results not shown). On the contrary, in the absence of salt, adsorption was improved, and both resins showed a promising pDNA binding profile (Figure 2A). In a prior study, Cruz et al. (2014) demonstrated that amino-positive groups in the agmatine structure facilitated electrostatic interactions with the negative phosphate groups in the pDNA molecule (Cruz et al., 2014). Regarding arginine-based chemistry prototypes, previous studies for pDNA purification revealed a complex interplay of interactions, including electrostatic forces, hydrophobic interactions, hydrogen bonding, and more, that may occur with the DNA bases or the DNA backbone (Sousa et al., 2008a). Among these, electrostatic interactions stand out as the major driving force due to the inherited negative charge of phosphate groups along the DNA backbone (Sousa et al., 2009a; Mahjoubin-Tehran et al., 2022). This elucidates the higher pDNA adsorption observed under low salt conditions, wherein electrostatic interactions are preponderant for promoting an effective binding.

Following these results, a more in-depth screening of conditions was performed by establishing the optimal parameters for pDNA adsorption, focusing on pH and contact time optimization.

To determine the optimal pH for pDNA adsorption, two pH values were tested in the buffers, namely, 100 mM Tris-HCl at pH 6 and Tris-HCl at pH 8. When the pH values were changed, the behavior of prototypes also varied. Prototype 1 tends to favor pDNA adsorption at pH 8, being able to capture approximately 9.4 µg pDNA/mg resin, in these conditions. However, reports suggest that lower pH levels may positively affect pDNA adsorption. This is because the protonation of amino groups is significantly affected by pH, and increasing the pH level results in weaker electrostatic interactions (Cruz et al., 2014; Bicho et al., 2016). In this context, some hydrophobic interactions can also contribute. The presence of a 3-carbon aliphatic chain within the agmatine structure may allow the establishment of hydrophobic interactions, influencing the adsorption of pDNA, particularly at pH 8 and synergically with electrostatic interactions. In comparison, prototype 14 performs better with lower pH levels, increasing pDNA adsorption to approximately double compared to pH 8 (Figure 2A). Indeed, previous research exploring the impact of pH on the interaction between pDNA and arginine has revealed that optimal adsorption occurs at lower pH levels, specifically around 6 and 7 (Cardoso et al., 2022).

To evaluate the impact of contact time, the sample was gently agitated while in contact with the resin for two distinct periods: 20 and 120 min. As predicted, a longer contact time led to increased pDNA adsorption. Figure 2B illustrates the contrast in pDNA adsorption between prototype 1 after 20 min and 120 min of contact time. In the case of prototype 14, there was no significant increase in adsorption (±0.2 μg/mg) when increasing the contact time from 20 to 120 min (results not shown).

## Screening of desorption conditions

**FIGURE 3:**
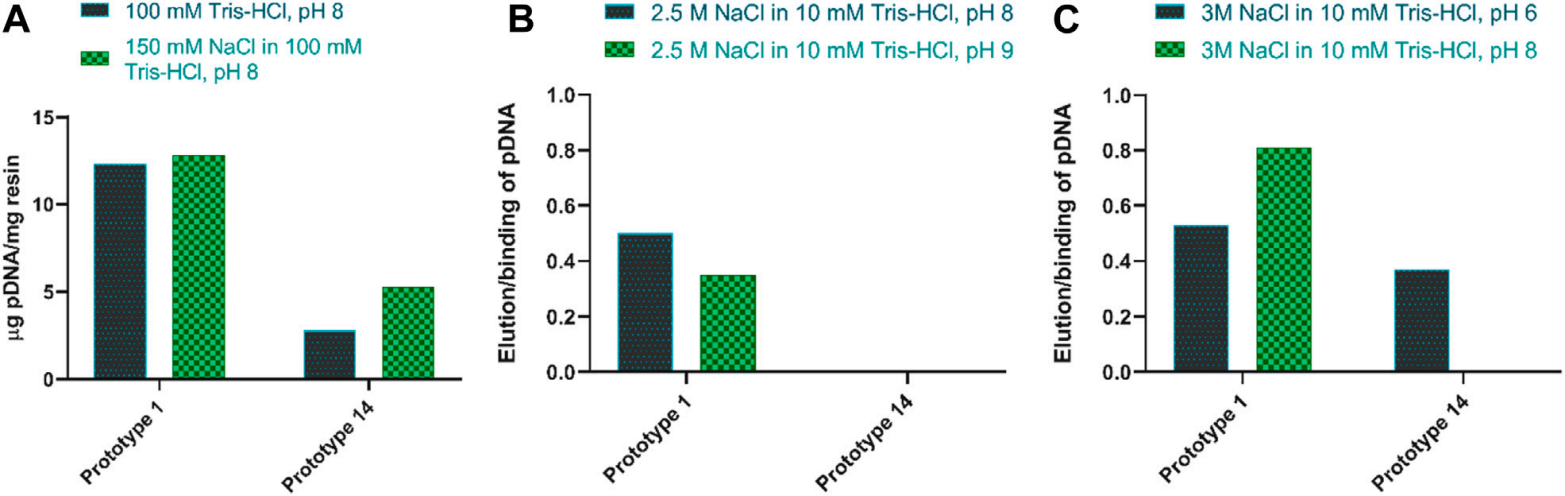
**(A)** Amount of pDNA adsorbed per mg of resin when adding 150 mM NaCl to the equilibration buffer, for both prototypes. **(B)** The ratio of elution per binding of pDNA when comparing 2.5 M NaCl in 10 mM Tris-HCl at pH 8 and pH 9, for both prototypes. **(C)** The ratio of elution per binding of pDNA when comparing 3 M NaCl in 10 mM Tris-HCl at pH 6 and pH 8, for both prototypes.

The desorption behavior was evaluated by altering the adsorption/elution buffer conditions and the contact time between the resin and the elution buffer. The desorption of pDNA, when promoting electrostatic interactions, is mainly attained by increasing the ionic strength in the elution buffer (Wan, 2021). An initial attempt was made to elute pDNA with 1.5 M NaCl in 10 mM Tris-HCl, pH 8.0. However, it resulted in poor pDNA recovery (<15%). As such, and to decrease the strength of the interaction between pDNA and the resin, 150 mM NaCl was added to the equilibration buffer, which increased the ionic strength in the binding conditions. As shown in Figure 3A, this did not compromise pDNA adsorption, but it did not improve the recovery, as well. However, for prototype 14, the adsorption improved with the addition of NaCl (Figure 3A). Salt supplementation to the mobile phase, when promoting electrostatic interactions, is a useful approach to remove impurities, as it can help avoid unspecific binding (Alves et al., 2021), what was already thoroughly studied in other works (Sousa et al., 2008a; Ebrahimpour et al., 2010). Therefore, the equilibration and binding condition were maintained with 150 mM NaCl. To enhance desorption efficiency, the subsequent steps involved assessing the impact of increasing NaCl concentration from 1.5 M to 2.5 M in the elution buffer while simultaneously testing two distinct pH values, pH 8 and pH 9. Although the elution values slightly increased at pH 8, they remained unsatisfactory when referring to both prototypes (Figure 3B). With these conditions, no desorption was achieved with prototype 14, and only around 50% recovery was attained for prototype 1. To improve the pDNA recovery, the NaCl concentration was further increased to 3 M, and the elution buffer was compared at pH 6 and 8. Notably, the most significant improvement was achieved when using a buffer of 3 M NaCl in 10 mM Tris-HCl at pH 8 as the elution buffer, with an elution ratio per binding of 0.81, for prototype 1 (Figure 3C). Since agmatine pKa is > 12.5 (Gründemann et al., 2003), higher pH values will decrease the strength of electrostatic interactions. Table 3 summarizes the best conditions achieved after the screening for optimal pDNA recovery.

**TABLE 3:** Summary of the established binding/elution conditions after the screening.

Resin	Binding conditions	Elution conditions	Contact time for elution (min)	pDNA (bound or recovered) (µg)	Elution/binding of pDNA
**Prototype 1**	150 mM NaCl, 100 mM Tris-HCl, pH 8	3 M NaCl, 10 mM Tris, pH 8	120	Bound: 10.8 Recovered: 8.8	0.81
**Prototype 14**	150 mM NaCl, 100 mM Tris-HCl, pH 8	3 M NaCl, 10 mM Tris, pH 6	120	Bound: 3.8 Recovered: 1.4	0.37

### Static binding capacity

**FIGURE 4:**
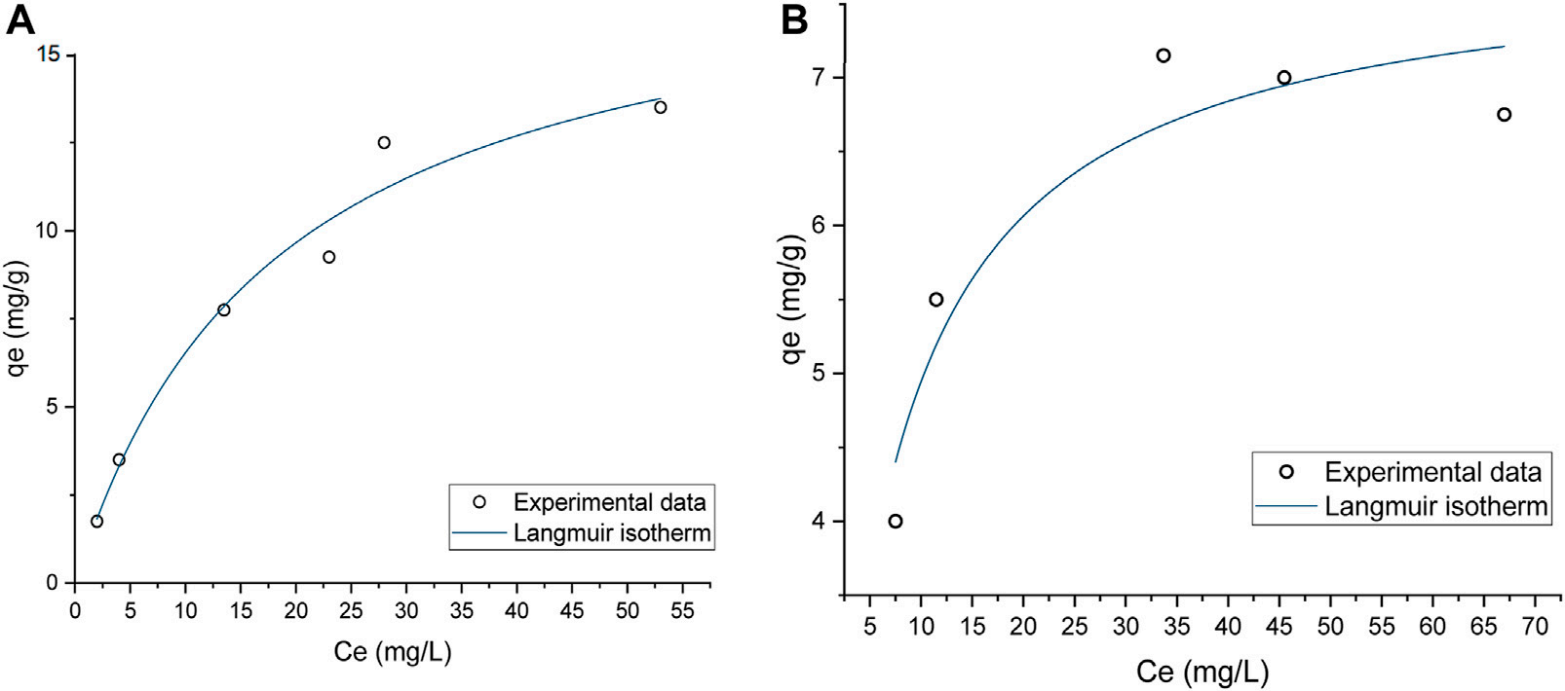
Static Binding Capacity of pDNA onto **(A)** Prototype 1, **(B)** Prototype 14, applying the Langmuir Isotherm.

Static binding capacity (SBC) assays were conducted to characterize the pDNA binding capacity. The data presented in Figure 4 shows that prototype 1 had the highest binding capacity, approximately 18.5 mg/g, indicating that it could be a promising resin for pDNA purification (Table 4). Also, it is possible to analyze the strength of the interaction between the adsorbate and the adsorbent by *K*_*L*_ (Groenenberg, 2018). A higher *K*_*L*_ value generally corresponds to a stronger interaction and a greater adsorption capacity. However, it is essential to note that this increased adsorption capacity can make it more difficult to recover the pDNA during elution. For example, if pDNA is adsorbed to a resin, a higher *K*_*L*_ value would likely result in a lower elution capacity. Prototype 1 has a lower *K*_*L*_ value of 0.055, meaning that besides having the best adsorption capacity, the pDNA could be eluted more easily compared to prototype 14. On the other hand, prototype 14 has a *K*_*L*_ of 1.71, which is significantly higher and could explain why it is more challenging to elute the adsorbed pDNA (as shown in topic 3.1.2). As for the maximum adsorption capacity in equilibrium, prototype 14 is significantly lower compared to prototype 1, only achieving approximately 7.842 mg/g (Table 4). The variation in ionic capacity can elucidate the disparity observed in the *q*_*max*_ values of the two prototypes. Considering the consistent particle size (50 µm) and identical matrix, based on polymeric beads, between agmatine- and arginine-based resin, the notable difference in ionic capacity becomes a pivotal factor in the analysis. As indicated in Table 2, the agmatine-based has a higher ionic capacity compared to the arginine resin. Consequently, it was expected that this would culminate in a superior maximum adsorption capacity when promoting electrostatic interactions, as verified.

**TABLE 4:** Maximum pDNA capacities *q*_*max*_ (mg pDNA per g of dry resin) and Langmuir constant *K*_*L*_ for both prototypes. Data correspond to the results presented in Figures 4A, B.

	*R* ^2^	*q*_*max*_ (mg pDNA per g of dry resin)	*K*_*L*_
**Prototype 1**	0.974	18.498 ± 2.20	0.055 ± 0.02
**Prototype 14**	0.903	7.842 ± 0.49	0.171 ± 0.50

Previous studies have shown that SBC for pDNA can vary depending on the type of chromatography used. For example, a study by Tarmann and Jungbauer, 2008 found that the SBC for pDNA on anion exchange columns can range from 1.3–13.4 mg/mL of column volume, which can be highly influenced by the specific conditions used, such as pH and ionic strength (Tarmann and Jungbauer, 2008). An example is the commercially available Fractogel^®^ EMD DEAE (M) resin, which is described to have a binding capacity of approximately 4.5 mg/mL, for pDNA, when supplemented with a salt concentration of 150 mM NaCl (KGaA, 2022). Another recent study is related to pDNA purification using arginine-agarose resin. This study evaluated pDNA adsorption at different pH values, reaching a maximum of 0.565 ± 0.033 mg/mL (Cardoso et al., 2022). Compared to these values, prototypes 1 and 14 yield a promising outcome by achieving a significantly higher SBC than a conventional anion exchange column.

### Gravity column assay

Batch assays serve as a valuable tool for the initial screening of adsorption conditions in pDNA purification. However, they may not fully encompass the diverse phenomena encountered in liquid chromatography processes. To achieve enhanced selectivity between sc and oc pDNA, and to gain deeper insights into the behavior during method scaling, a second screening of adsorption and recovery of pDNA was conducted. Gravity flow columns were packed with 100 mg of dry resin for each prototype, and the screening process was initiated based on the conditions previously established in the batch assays.

The adsorption conditions established earlier demonstrated a favorable binding capacity. Although further investigation revealed slight improvements for prototype 14, when using 100 mM phosphate buffer, pH 6.0 as binding condition. This adjusted condition led to slightly improved adsorption of pDNA (results not shown), and for that reason it was also considered in further experiments. Table 5 demonstrates the pDNA binding performance for both resins, effectively capturing nearly 50 µg. However, the elution step posed a significant challenge. Initially, using 3 M NaCl as an elution condition proved insufficient for effective pDNA recovery (results not shown). An alternative strategy encompassing an additional elution step was implemented to address this issue.

**TABLE 5:** Improved conditions for selective elution of sc pDNA from prototype 1 and prototype 14.

Resins	Binding conditions	1st elution condition	2nd elution condition	pDNA (bound and recovered) (µg)
**Prototype 1**	150 mM NaCl, 100 mM Tris-HCl pH 8.0	1.5 M NaCl +375 mM Arg, 100 mM Tris-HCl pH 8.0	3 M NaCl, 10 mM Tris-HCl pH 8.0	**Bound:** 48.0 **1st step Recovered:** 1.5 **2nd step Recovered:** 22.5
**Prototype 14**	100 mM Phosphate buffer pH 6.0	35 mM Arg, 100 mM Tris-HCl pH 8.0	3 M NaCl, 10 mM Tris-HCl pH 8.0	**Bound:** 44.5 **1st step Recovered**: 5.5 **2nd step Recovered:** 33.0

**FIGURE 5:**
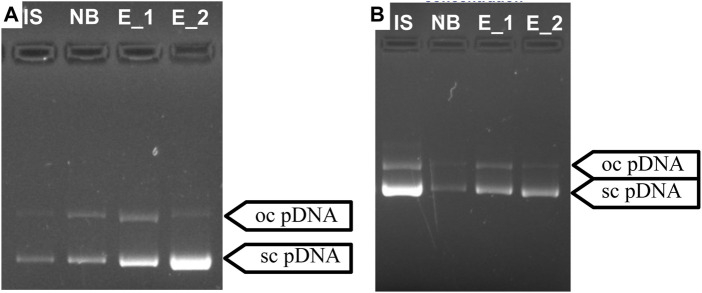
Agarose gel electrophoresis for the optimized separation of sc pDNA and oc pDNA for prototypes 1 **(A)** and 14 **(B)**. IS—initial sample; NB - Fraction of pDNA not bound to the column; E_1—1^st^ Elution step; E _2—2^nd^ Elution step.

In order to achieve a gradual desorption of pDNA, an intermediate step was included to simulate a gradient during the elution process. Moreover, following the approach studied and employed by Sousa et al. (2008a), Sousa et al. (2008b), arginine was incorporated into the buffer used in the first elution step (Sousa et al., 2008a). Arginine finds extensive utility across diverse chromatography methods. It is employed, for instance, in solubilizing and suppressing protein aggregates (Arakawa et al., 2007b; Arakawa et al., 2007c) and facilitating their effective elution from various column resins (Arakawa et al., 2007a; Arakawa et al., 2007c). Additionally, arginine serves as a competitive agent for pDNA elution (Sousa et al., 2009a). Some previous works described that arginine allows total elution of pDNA (Sousa et al., 2008a; Sousa et al., 2009b), improving the effectiveness of the elution compared to when using NaCl exclusively (Sousa et al., 2009a). Indeed, adding arginine for the purpose of this work proved highly beneficial, significantly enhancing the elution efficacy and facilitating a selective separation between oc pDNA and sc pDNA. The findings from this strategy align with the previous research, confirming that free arginine will bind preferentially to pDNA, weakening the interactions with the resin and improving its effectiveness as an elution optimization strategy (Sousa et al., 2008a). Among the methods tested for prototype 1, the most promising approach involved adding 1.5 M NaCl and 375 mM arginine to the 1st elution buffer. Besides successfully achieving total pDNA binding (48 µg), this method also allowed a recovery yield of approximately 50% (Table 5). Notably, Figure 5A reveals that almost exclusively sc pDNA was recovered in the final step of elution. For prototype 14, the optimal method entailed the addition of 35 mM arginine to the first elution buffer. This approach also allowed good pDNA binding and recovery, as shown in Table 5. More importantly, both established methods achieved remarkable selectivity, with sc pDNA being predominantly recovered in the last step of the elution process (Figure 5B). Selectivity is achieved by exploiting the structural differences between sc and oc conformations. The compact nature of sc pDNA characterized by the lower surface area and higher exposure of nucleotide bases, increases the strength of the interaction to the amino groups from both resins, surpassing that observed with the oc isoform (Cruz et al., 2013). These findings underscore the potential of both resins to be explored as chromatographic media for pDNA polishing.

### Chromatographic assays

**FIGURE 6:**
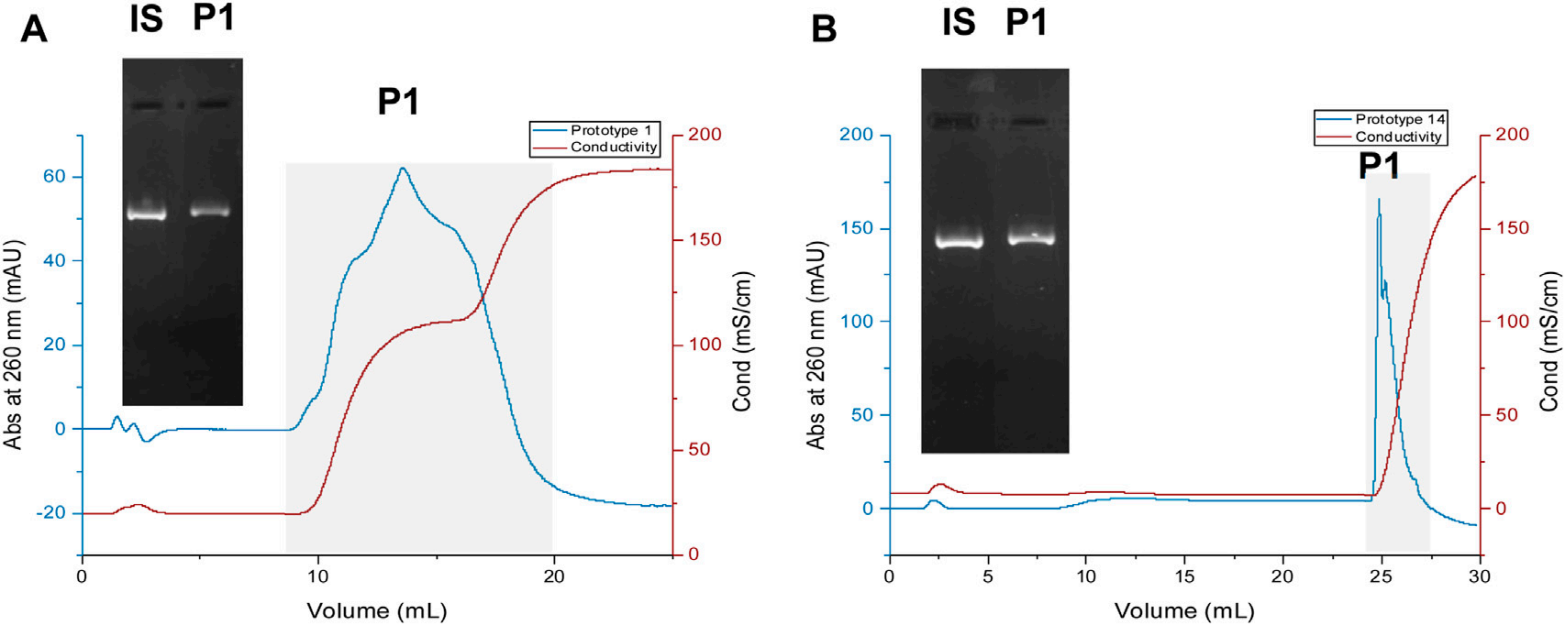
Chromatographic profiles of pDNA loaded onto prototypes 1 **(A)** and 14 **(B)**. IS—initial sample. For the agarose gel electrophoresis, the fractionated sample identified as “P1” was collected, desalted, and concentrated to 200 µL for both assays, following the procedure described in the “Methods” section.

Chromatographic assays were finally performed to evaluate the behavior and characterize each resin’s chromatographic profile when working with a steady flow rate of 1 mL/min. A total bed volume of 1 mL was packed for each prototype, and the solutions utilized in this method are detailed in Table 5. A sample obtained from an *E. coli* lysate followed by a capture step was utilized to ensure that the prototypes were evaluated under realistic conditions. Figure 6 illustrates the chromatographic profile of prototype 1 (Figure 6A) and prototype 14 (Figure 6B), and the respective agarose gel electrophoresis of the initial sample and the sample collected from the peak, represented as P1. Initial analyses of both prototypes revealed that the injected pDNA sample effectively bound and eluted from each resin, using the established conditions, even when dealing with more complex samples.

Regarding prototype 1, the elution of pDNA begins during the first elution step. However, due to the high concentration of L-arginine used for promoting elution in this step, the chromatographic profile of the eluted peak appears inaccurate. The slight broad peak arises because L-arginine can absorb at the same wavelength as pDNA (260 nm) (Kumar and Rai, 2010). Consequently, throughout the 1^st^ elution step, L-arginine interferes with the chromatogram of the eluted pDNA. Nevertheless, the corresponding agarose electrophoresis of the eluted peak (Figure 6) confirms that the injected sample is eluting.

Regarding prototype 14, pDNA elution is not evident until the 2^nd^ elution step. However, when increasing the ionic strength, in the second elution buffer, containing 3 M NaCl, the elution of pDNA occurs, and a narrow peak is obtained. The capture of the entire pDNA sample on the resin was observed upon injection, and only when applying a high-salt concentration pDNA eluted, suggesting the robust interaction between pDNA molecule and resin.

In each assay, the peak labeled as P1 (Figure 6) was collected and subjected to comprehensive characterization, encompassing sc pDNA quantification, recovery assessment, % purity, and impurity analysis. Additionally, an evaluation of Dynamic Binding Capacity (DBC) was carried out to determine the capacity of each resin. These analyses provide valuable insights into the performance and suitability of the chromatographic process for sc pDNA polishing.

### Quantitative analysis of sc pDNA recovery and purity

The quality and quantification of sc pDNA in the fractionated pool were subjected to further evaluation using analytical chromatography. It was employed a TSKgel^®^ DNA-NPR column based on weak anion exchange chromatography, known for its ability to separate different pDNA isoforms. By constructing a calibration curve (Supplementary Figure S1), sc pDNA quantification was possible, enabling precise measurements of its purity and recovery yield. To confirm and validate the peak corresponding to oc pDNA, a pDNA sample was digested by the restriction enzyme Nt.BspQI. Nt.BspQI is an enzyme that cuts specifically one strand of DNA on a double-stranded DNA substrate, converting the sc pDNA isoform into oc pDNA. The retention time from the peak related to the oc pDNA was compared with the chromatographic profile of the complex pDNA sample, and the oc pDNA peak was confirmed.

The data presented in Table 6 indicates that Prototype 14 significantly outperforms Prototype 1 regarding pDNA recovery, with a recovery yield of 52.2%, far surpassing the 10.09% achieved by Prototype 1. The strong interaction established between agmatine and pDNA could be responsible for the low recovery attained, which can also be correlated with the high ionic capacity (691 µeq/g resin) of the agmatine-based resin. This phenomenon corroborates the difficulties of achieving high recovery and elution, also referred by Matos et al. (2014) and Bicho et al. (2016). Nonetheless, as demonstrated previously, the potential to achieve selectivity can be achieved, and the binding capacity can be elevated. This, in turn, translates to a reduced requirement for resin mass to capture a given quantity of pDNA, as demonstrated in previous findings. Furthermore, both prototypes exhibited promising results when assessing the purity of each fractionated peak relative to oc pDNA, boasting relative purity levels exceeding 93%.

**TABLE 6:** Analytical analyses of purity and recovery yield of the sc pDNA isolated with Prototype 1 and Prototype 14.

Resin	Initial sample (µg)	Recovered (µg)	% recovery	% purity (relative to oc pDNA)
**Prototype 1**	26.36	2.66	10.09	93.54
**Prototype 14**	26.36	13.76	52.20	95.90

Despite the high purity levels achieved, it is important to note that the lower recovery yield can be attributed to several factors that primarily arise from the elution conditions. It is crucial to understand that purity and recovery are interconnected and optimizing one may inadvertently impact the other. Hence, going forward, it is imperative to conduct a thorough assessment of each variable to improve the recovery yield non disregarding the purity of pDNA.

### Quantitative analyses of the main impurities

A more detailed characterization of the sc pDNA recovered after each resin assay was carried out, encompassing measurements of endotoxins, proteins, and gDNA, which were then compared to the initial feed, prior to the purification step with the resins. The results described in Table 7 revealed a decrease in impurities levels. The microassay procedure revealed that protein levels were undetectable in both the initial and fractionated samples (results not shown). Endotoxin content remained unchanged before and after the purification procedure for both prototypes. However, a significant reduction in gDNA level was observed after the chromatographic assays. For prototype 1, the gDNA level decreased from 81.6 µg to 3.6 µg, while for prototype 14, it was reduced to 14.8 µg. This translates to more than 95% reduction on gDNA levels when referring to prototype 1 and more than 81% for prototype 14. These findings indicate the effectiveness of both prototypes in removing gDNA during the chromatographic process, contributing to the enhanced purity and quality of the recovered sc pDNA.

**TABLE 7:** Analyses on the levels of the main impurities derived from *E. coli* recombinant production (gDNA, proteins, and endotoxins).

	Main impurities	
	gDNA (µg)	Endotoxins (EU)
Initial sample	81.6	0.50
Prototype 1	3.6	0.56
Prototype 14	14.8	0.54

### Dynamic binding capacity for pDNA

DBC was assessed by quantifying the amount of pDNA that can be loaded onto the column. The DBC value can vary based on several factors, including the type of column, the size and charge of the pDNA, and the specific purification conditions employed. In these assays, the optimal conditions detailed in Table 5 were utilized for the adsorption of pDNA to ensure accurate DBC determination. This critical parameter sheds light on the resin’s ability to bind pDNA effectively during the chromatographic process, which is vital for designing efficient and scalable purification protocols and taking advantage of the robustness of the resins. When comparing prototypes 1 and 14, it was verified that prototype 1 exhibits a substantially higher capacity than prototype 14. At 10% breakthrough, prototype 1 reached a 6.099 mg pDNA/mL prototype, while prototype 14 only achieved a DBC of 1.148 mg pDNA/mL prototype (Table 8).

**TABLE 8:** Dynamic binding capacity of prototype 1 and prototype 14. Experiments were performed at 0.5 mL/min with a 25 μg/mL pDNA sample, and capacity was estimated at 10% and 50% breakthrough.

Breakthrough (%)	Flow rate (mL/min)	DBC (mg pDNA/mL prototype)	
		Prototype 1	Prototype 14
10	0.5	6.099	1.148
50	0.5	13.970	5.563

Numerous studies have reported a variety of strategies for pDNA purification, each associated with a respective DBC. For instance, in 2016, an agmatine monolithic disk was evaluated by exploiting hydrophobic interactions. At a flow rate of 1 mL/min, using a plasmid sample with a concentration of 25 μg/mL, a DBC of 1.040 mg pDNA/mL was achieved at 10% breakthrough. On the other hand, when operating at a flow rate of 0.5 mL/min resulted in a DBC of 5.603 mg pDNA/mL at 10% breakthrough (Bicho et al., 2016). In a separate study by Valente et al. (2020), a method involving arginine-modified microporous support was evaluated. Employing a 0.5 mL/min flow rate and a pDNA concentration of 50 μg/mL, the DBC reached 0.256 mg pDNA/mL (Valente et al., 2020). Regarding commercially available resins, it is reported that Fractogel^®^ EMD DEAE presents an adsorption capacity of 2.45 mg pDNA/mL (Eon-Duval and Burke, 2004), CIM C4 HLD presents an adsorption capacity of ≥2.5 mg pDNA/mL (Smrekar et al., 2010), and CIM DEAE presents an adsorption capacity of 13.42 mg pDNA/mL (Tarmann and Jungbauer, 2008). By comparison, both prototypes evaluated in this work showed similar or higher binding capacities, even when compared with commercially available resins. These results further reinforce the great potential of agmatine- and arginine-based resins for pDNA purification/polishing.

## Conclusion

The demand for pDNA-based therapies highlights the need to develop effective purification strategies that preserve its integrity and biological activity. The chromatography process can be influenced by the choice of support and ligand chemistries, affecting the efficiency, robustness, and specificity of the purification process.

Evidence from the study highlights prototype 1 (agmatine-based resin) and prototype 14 (arginine-based resin) as promising candidates for sc pDNA purification. Amino acids, such as these two prototypes, have been arising as a good solution for a selective and specific biorecognition of the sc pDNA. They mimic the interaction that occurs naturally in biological organisms, leading to a multitude of interactions that can be exploited between pDNA and the resins, resulting in a high adsorption capacity. In this regard, prototype 1 achieved approximately 57% higher SBC than prototype 14, and approximately 81% higher DBC. However, due to the strong interactions, eluting the pDNA from prototype 1 was more challenging, resulting in a lower recovery (10.09%). On the other hand, prototype 14 achieved a good recovery yield (>50%). This goes to show that there is a fine line between selectivity and capacity, which usually have opposite tendencies, as explained by Valente et al. (2021). Further optimization on the ligands density could improve the recovery of pDNA. Also, to avoid and mitigate the possible column poisoning due to the strong interactions, it is important to proper precondition the column, by performing regeneration between assays.

Disregarding the strength of interactions, both prototypes showed excellent and promising prospects for the selective polishing of sc pDNA. Indeed, both prototypes were able to significantly reduce the amount of gDNA present in the pDNA sample after the capture step. Ultimately, both prototypes showed a purity above 93% relative to the oc pDNA isoform. Nonetheless, it is important to note that the endotoxins content was not altered in this step, but their levels were previously reduced in the capture step. Anyway, this should also be considered from the point-of-view of the potential therapeutic application of pDNA.

Overall, this screening process provided valuable insights into the behavior of different resins concerning pDNA adsorption and elution, as well as establishing optimal conditions for the purification of sc pDNA for the most promising prototypes. Additionally, some tested resins showed promise for pDNA purification comparable to, if not better, commercially available resins.

## Data Availability

The datasets presented in this article are not readily available because Datasets are subjected to confidentiality. Requests to access the datasets should be directed to fani.sousa@fcsaude.ubi.pt.
