# Supplementary material for: Evaluation of novel chromatographic prototypes for supercoiled plasmid DNA polishing
Source: Front Bioeng Biotechnol. 2024 Jan 4;11:1296444. doi: 10.3389/fbioe.2023.1296444 (PMC10797707; doi:10.3389/fbioe.2023.1296444)
Supplement: Supplementary file 1 [file DataSheet1.docx]

Evaluation of novel chromatographic prototypes for supercoiled plasmid DNA polishing

Pedro L. Ferreira^1^, Helena Marie^2^, Tim Berger^2^, Bianca Edelmann^2^, Oliver Rammo^2^, Fani Sousa^1,*^

^1^CICS-UBI – Health Sciences Research Centre, University of Beira Interior, Av. Infante D. Henrique, 6200-506 Covilhã, Portugal

^2^Merck Life Science KGaA, Frankfurter Str. 250, 64293 Darmstadt, Germany

*** Correspondence:**Fani Sousa
[fani.sousa@fcsaude.ubi.pt](mailto:fani.sousa@fcsaude.ubi.pt)

Supplementary Material

# Supplementary Figures

## Quantitative analysis of sc pDNA recovery and purity

Supplementary Figure 1. (A) Chromatographic profiles of each point of the calibration curve. (B) sc pDNA calibration curve, ranging from 2.5 µg/mL to 200 µg/mL. (C) Chromatographic profile of oc pDNA overlapped with the chromatographic profile of a pDNA sample containing both oc and sc isoforms.
